# Abdominal pain in an adult with Type 2 diabetes: A case report

**DOI:** 10.1186/1757-1626-1-154

**Published:** 2008-09-17

**Authors:** George Panagoulias, Nicholas Tentolouris, Spiros S Ladas

**Affiliations:** 1 1st Department of Propaedeutic Medicine, Athens University Medical School, Laiko General Hospital, Athens, Greece

## Abstract

**Introduction:**

Chronic abdominal pain (CAP) may be a manifestation of diseases involving many intra-abdominal organs. Beside diseases affecting subjects without diabetes mellitus, diabetic patients may have CAP due to diabetes-related complications like neuritis, motor diseases of the gastrointestinal tract and autonomic dysfunction. Atherosclerosis is 2–4 times more common in patients with diabetes and affects mainly carotid, coronary, iliac and lower limb arteries as well as aorta. Another less common complication is chronic mesenteric ischemia (CMI, intestinal angina), caused by atherosclerotic obstruction of the celiac artery and its branches and results in episodic or constant intestinal hypoperfusion.

**Case presentation:**

We present a case of a diabetic patient with CMI in whom the diagnosis was delayed by almost 5 years. The dominant symptoms were crampy abdominal postprandial pain, anorexia, changes in bowel habits and cachexia. Conventional angiography revealed significant stenosis of the celiac artery and complete obstruction of the inferior mesenteric artery. Noteworthy, no significant stenoses in carotids or limbs' arteries were found. Revascularization resulted in clinical improvement 1 week post-intervention.

**Conclusion:**

CAP in patients with diabetes may be due to CMI. The typical presentation is crampy postprandial abdominal pain in a heavy smoker male patient with long-standing diabetes, accompanied by anorexia, changes in bowel habits and mild to moderate weight loss. At least two of the three main splanchnic arteries must be significantly occluded in order CMI to be symptomatic. The diagnostic procedure of choice is conventional angiography and revascularization of the occluded arteries is the radical treatment.

## Introduction

Chronic mesenteric ischemia (CMI) was first described in 1894 by Councilman. CMI, also called intestinal angina, is caused by episodic or constant intestinal hypoperfusion due to mesenteric atherosclerotic disease, vasospasm and/or hypoperfusion of the mesenteric vasculature. Patients with CMI often present with subtle or non-specific symptoms; physical findings may be absent. CMI can worsen and develop into acute intestinal ischemia with bowel infarction, leading to sepsis and death if diagnosis delays. We describe herein a patient with type 2 diabetes mellitus with CMI in whom the diagnosis was delayed by almost 5 years. Noteworthy, no significant stenoses in the carotid or leg arteries were found by Duplex and Triplex ultrasonography.

## Case presentation

A 57-year-old Caucasian male, heavy smoker patient with type 2 diabetes mellitus diagnosed 15 years ago and treated with insulin during the last 4 years, was admitted to the hospital because of mild constant abdominal pain, which was exaggerated after eating and drinking water. The pain started some 5 years ago, gradually worsened, and was accompanied by reluctance to eat, weight loss, nausea, vomiting and changes in bowel habits. On admission, the patient was cachectic and vital signs were in the lower normal values. On physical examination, diffuse abdominal pain in both superficial and deep palpation was noticed. The patient had background diabetic retinopathy, proteinuria due to diabetic nephropathy and peripheral neuropathy. He has been treated with isophane insulin 15 IU in the morning and 12 IU in the evening, pentoxifylline 300 mg bid and ramipril 5 mg once a day. His HbA1c was 7.1%. The patient did not have diabetic ketoacidosis or hyperglycaemic hyperosmolar state during his hospitalisation, and thus pseudoperitonitis diabeticorum, a complication which may accompany these metabolic disturbances, was ruled out. Spinal mono- or polyradiculopathy was excluded by detailed history, clinical examination and paraspinal electromyography. The patient underwent endoscopy of the upper and lower gastrointestinal tract to exclude oesophageal, stomach or large bowel disease, but the examination was unremarkable. Helicobacter Pylori was not detected on biopsies obtained from the stomach. Abdominal ultrasound examination, imaging with computerized tomography (CT) of the abdomen and magnetic resonance imaging (MRI) of the pancreas were normal. Immunological studies for vasculitis were negative. Other causes of abdominal pain, such as aortic aneurysm, hepatitis, cholecystitis, pancreatitis and splenic infarctions where ruled out by paraclinical work-up. A presumptive diagnosis of arterial occlusive disease was made and the patient underwent invasive catheter celiac arteriography.

Angiography revealed 90% stenosis of the celiac artery trunk (CA) and total occlusion of the inferior mesenteric artery (IMA). Thus, the intestinal angina was diagnosed. The patient underwent percutaneous transluminal mesenteric angioplasty (PCTA) and stent insertion in the CA. Surgical revascularization of the IMA was not possible. The abdominal pain gradually improved 1 week post-revascularization intervention. Two months later he was free of pain and gained 2 kg in weight. Further examination by Doppler and Triplex ultrasonography did not reveal heamodynamically significant stenoses in the carotid and lower limb arteries.

## Discussion

Chronic pain is usually defined as pain which lasts beyond the ordinary duration of time that an insult or injury to the body needs to heal. The pain is defined as chronic when its duration lasts for 3 to 6 weeks; when the duration is shorter the pain is considered as acute. The difference between acute and chronic pain is of great importance, since acute pain may be a harbinger of a serious condition and delays in the diagnosis and treatment may lead to unfavourable outcome [1].

Patients with diabetes, like elderly and immunocompromised patients, may present with less common causes of abdominal pain. For mnemonic reasons it is easier to consider causes of abdominal pain following anatomical sequence from head to toe at first and to consider causes of pain found in special populations and rare causes, secondly.

To begin with, diabetic patients may present with pain perceived as originating in the abdominal area while the primary disease is located in extra-abdominal sites. Such an example is neuropathic pain due to post-herpes zoster virus infection involving the thoracic dermatomes (post-herpetic neuralgia), which may be misdiagnosed if history and clinical examination is not detailed [2]. Angina pectoris may present as CAP, especially postprandially. Pneumonia involving the lower lobes of the lung, accompanied or not by pleuritic effusion, may be another cause of abdominal pain, presumably related to diaphragmatic irritation [3].

Gastrointestinal (GI) symptoms are more common in patients with diabetes and often reflect diabetic GI autonomic neuropathy [4]. However, symptoms may be more likely due to other factors than to autonomic dysregulation [5]. Esophageal dysfunction, defined as disordered peristalsis and/or abnormal lower esophageal sphincter function, is a common cause of postprandial upper abdominal pain. Gastroparesis diabeticorum, accompanied with pylorospasm, may be another cause of upper abdominal pain in the postprandial state; nausea, vomiting, early satiety and fullness, anorexia and epigastric discomfort often accompany this complication. In such cases peptic ulcer disease and its complications, as well as pyloric stenosis, must be considered in the differential diagnosis and upper gastrointestinal endoscopy is warranted. Delay in gastric emptying after consumption of radiolabeled solid food and manometric studies confirms diagnosis of gastroparesis diabeticorum [6]. Dyspepsia is defined as persistent or recurrent abdominal pain or discomfort in the upper abdomen. Dyspepsia is common in the general population and diagnosis is made when other causes of abdominal pain are excluded [6-8].

Gallbladder atony and enlargement is often found in diabetic patients [5]. Cholelithiasis, biliary dyskinesia, prolonged or recurrent cystic duct blockage leading to cholangitis, inflammation, or infection, must be considered in the differential diagnosis. Long-term treatment with third generation cephalosporins may result in gallstones formation [9]. Diabetic patients who are hospitalised for diabetic foot or other chronic infections may be treated with such medications for weeks or even longer and are more susceptible to gallstone formation than non-diabetic patients. Hepatitis, splenic abscesses and infarctions are easily ruled out by laboratory work-up and imaging studies of the abdomen [10,11]. Chronic pancreatitis and the complications of acute pancreatitis (e.g. abscesses and pseudocysts) may cause CAP with the same frequency as in the general population [12,13].

Inflammatory bowel disease should be included in the differential diagnosis and upper and lower GI tract endoscopy must be performed when concomitant symptoms, apart from pain, exist [14,15]. Chronic appendicitis is often under diagnosed and is often accompanied with fever and leukocytosis. Some patients diagnosed with chronic appendicitis actually appear to have recurrent acute appendicitis. Pain is usually localized to the right lower quadrant when the appendiceal inflammation begins to involve the peritoneal surface. However, when the appendix is rear, only digital rectal examination may confirm the diagnosis [16]. Diverticular disease and diverticulosis are often asymptomatic and may be found incidentally during colonoscopy or sigmoidoscopy performed for other reasons. However, some patients with diverticular disease complain of mild abdominal symptoms, mainly crampy pain. Thus, in case of a patient with CAP, diverticular disease must be ruled out by lower GI tract endoscopy [17]. Nephrolithiasis may present as chronic intermittent pain, especially if the patient is not able to provide a good medical history. The location of the pain may change as the stone migrates. A urine specimen usually shows haemoglobin or red blood cells; ultrasonography usually shows the stone or pelvicaliceal distension [18].

Patients with abdominal aortic aneurysm can present with diffuse or localized abdominal pain. Patients with type 2 diabetes mellitus have often atherosclerosis and are more susceptible to development of abdominal aortic aneurysm [19]. Diagnosis is easily made by ultrasound examination of the abdominal aorta or CT of the abdomen. Patients with infectious diseases causing CAP, such as tuberculosis, yersiniosis and Whipple's disease may present with abdominal wall rigidity due to activation of primary afferent visceral and cutaneous pain nerves [20,21]. Helminthic and other tropical infectious diseases should also be considered in patients with epidemiological history [22]. Diabetic patients with advanced renal stage and those under haemodialysis are more susceptible to such infections [21]. Partial intestinal obstruction due to incarcerated hernia, intra-abdominal adhesions and partial intussusception usually result in acute abdominal pain [23].

Chronic pelvic pain in men is due either due to prostatodynia or to bacterial prostatitis [24]. Lower abdominal pain in women is frequently caused by disorders of the internal reproductive organs. A pelvic inflammatory process may progress to chronic pelvic pain in a percentage up to 30% [25]. Endometriosis is common in women suffering from chronic pelvic pain and it is the most common diagnosis made at the time of gynaecological laparoscopy [26]. Adenomyosis, swelling of endometrial islands and the ovarian remnant syndrome although are rarely manifest with pain only, should be considered in the differential diagnosis of chronic pelvic pain in women [27]. Pelvic organ prolapse usually is a sensation of pelvic pressure and/or heaviness or protrusion of tissue from the vagina. However it is rarely manifested as pain only and diagnoses is easy to be made by rectovaginal examination [28].

Patients with human immunodeficiency virus (HIV) infection and acquired immunodeficiency syndrome (AIDS), with or without diabetes, are often referred for GI and hepatobiliary symptoms, including pain. In the majority of such cases abdominal pain is directly related to HIV infection and to GI tract involvement [29]. Patients with coagulopathies along with diabetes, may spontaneously develop hematomas on the bowel wall and thus abdominal pain [30]. Patients with sickle cell disease and diabetes, apart from acute abdominal pain as part of a vaso-occlusive crisis, may present with chronic pain, due to diseases in the setting of hepatic involvement, complications of cholelithiasis, splenic infarction, ischemic colitis, and non-surgical genitourinary disorders [31,32].

Even more rare causes of abdominal pain should be considered in diabetic patients with CAP when the cause is not clear [33]. Painful rib syndrome is a benign condition characterized by discomfort in the lower chest or upper abdomen, tenderness over the costal margins, and reproduction of the pain by pressure on the ribs and it is most common in women [34]. Wandering spleen syndrome's complication, if the underlying disease is not diagnosed on time, is another cause of chronic pain especially in young adolescents. Abdominal imaging confirms the diagnosis by showing splenic infractions due to absence of splenic blood flow due to torsion [35]. Abdominal migraine is a rare entity diagnosed in patients who usually also suffer from typical migraine headaches, although occasionally patients present with gastrointestinal symptoms only [36]. Eosinophilic gastroenteritis has to be ruled out in patients with abdominal pain, nausea, vomiting, and diarrhoea. The signs and symptoms are related to the organs and to the extend that are being infiltrated be eosinofils and the ddiagnosis is established by histopathological examination of tissue biopsy [37]. The Fitz-Hugh-Curtis syndrome or perihepatitis due to post-Chlamydia trachomatis or to Neisseria gonorrhoea infection may be a rare cause of right upper quadrant pain in young women [38]. Hereditary angioedema is caused by abnormal activation of the complement-mediated inflammatory pathways due to defects in the C1 inhibitor of the classical complement pathway. It and can present usually in adolescence with recurrent episodes of abdominal pain, accompanied by nausea, vomiting, colicky pain, and diarrhoea, apart from swelling of other tissues and life-threatening laryngeal swelling. Episodes of abdominal pain resolve spontaneously in two to four days [39]. Abdominal malignancies or metastatic disease to abdominal organs are also other causes of abdominal pain. Finally, abdominal pain may be psychogenic in origin and somatization, apart from irritable bowel syndrome, may cause recurrent chronic abdominal pain; psychogenic pain is more common in adolescents [40].

To come to chronic mesenteric ischemia, this clinical entity merits consideration in elderly patients presenting with chronic diffuse abdominal pain. CMI is defined as postprandial abdominal pain with weight loss and anorexia. Changes in bowel habits, nausea and vomiting, postprandial angina, anorexia, gastrointestinal bleeding, and systemic signs such as fever and hypotension, are less common. Pain is typically crampy, localised in the upper abdomen and appears 10–15 minutes postprandially. The pain steadily increases, plateaus and resolves 1–4 hours after food consumption [41]. Weight loss can average 10 – 15 kg and is primarily the result of reduced food intake in order to avoid intestinal angina. Abdominal angina is caused by repeated transient episodes of inadequate intestinal blood flow after meals [42]. Chronic dull abdominal pain develops as the occlusion progresses [43]. The main cause of CMI in more than 90% of the cases is atherosclerosis of the celiac artery (CA). The well-known risk factors for atherosclerosis are implicated in the development of CMI. Other less common non-atheromatous causes of CMI may be Takayasu arteritis, dysplastic lesions, thromboangiitis obliterans, radiation-induced vascular injury and external pressure from intra-abdominal masses, and primary or secondary thrombosis of the mesenteric venous system. Physical examination and laboratory tests rarely contribute to diagnosis. A bruit at the epigastric area may be the only clue to the diagnosis but it is not common [41]. Many diagnostic algorithms [41,42,44] and tests including conventional interventional angiography, duplex ultrasonography, computerised tomography angiography and magnetic resonance angiography, have been proposed for the diagnosis of CMI, but none has proven to be sufficiently sensitive or specific [42]. Provocative balloon tonometry is the only test for the evaluation of the adequacy of intestinal blood flow [44]. The "gold standard" diagnostic procedure for CMI remains conventional angiography. At least two out of the three celiac arteries are usually found to be totally occluded or with severe stenosis in the vast majority of most patients suffering from CMI. Patients with such symptoms should be suspected of having CMI, unless other causes of abdominal pain are found. Diagnosis is based on clinical symptoms and angiographic evaluation of the splanchnic arteries, after exclusion of other gastrointestinal diseases. According to current guidelines and consensus statement, patients with intestinal angina with few risk factors should be treated by surgical revascularization (endarterectomy or bypass grafting) [45]; patients at higher risk should be managed by PCTA with or without stenting [42,46]. Other revascularization procedures include transaortic endarterectomy and antegrade visceral bypass [45].

## Conclusion

Chronic abdominal pain (CAP) is not rare and may be a manifestation of diseases involving many intra-abdominal organs. Apart from diseases affecting primarily the stomach, liver, biliary tract, pancreas, kidneys and intestine, advanced atherosclerotic lesions of the celiac artery may cause mesenteric ischaemia and chronic abdominal pain. The classic profile of a patient with CMI is that of a male heavy smoker with postprandial abdominal pain, sitophobia, and mild to moderate weight loss. At least two of the three main splanchnic arteries must be significantly occluded in order CMI to be symptomatic. A high degree of clinical suspicion for the disease is needed and the diagnostic procedure of choice is conventional angiography. Revascularization of the occluded arteries is the treatment of choice.

## Competing interests

The authors declare that they have no competing interests.

## Authors' contributions

GP undertook writing and the literature review and submitted the article. NT undertook the literature and contributed to the writing and literature review. GP, NT and SDL were responsible for diagnosis, patient management and review. All authors read and approved the final manuscript.

## Consent

Written informed consent was obtained from the patient for publication of this case report and any accompanying images. A copy of the written consent is available for review by the Editor-in-Chief of this journal.

## References

[B1] Woolf CJ (2004). Pain: moving from symptom control toward mechanism-specific pharmacologic management. Ann Intern Med.

[B2] Schmader K, Gnann JW, Watson CP (2008). The epidemiological, clinical, and pathological rationale for the herpes zoster vaccine. J Infect Dis.

[B3] Kass SM, Williams PM, Reamy BV (2007). Pleurisy. Am Fam Physician.

[B4] Vinik AI (1999). Diagnosis and management of diabetic neuropathy. Clin Geriatr Med.

[B5] Vinik AI, Maser RE, Mitchell BD, Freeman R (2003). Diabetic autonomic neuropathy. Diabetes Care.

[B6] Talley NJ (2007). Functional gastrointestinal disorders in 2007 and Rome III: something new, something borrowed, something objective. Rev Gastroenterol Disord.

[B7] Delaney B, Ford AC, Forman D, Moayyedi P, Qume M (2005). Initial management strategies for dyspepsia. Cochrane Database Syst Rev.

[B8] Talley NJ, Zinsmeister AR, Schleck CD, Melton LJ (1992). Dyspepsia and dyspepsia subgroups: a population-based study. Gastroenterology.

[B9] Acun C, Erdem LO, Sogut A, Erdem CZ, Tomac N, Gundogdu S (2004). Ceftriaxone-induced biliary pseudolithiasis and urinary bladder sludge. Pediatr Int.

[B10] Nores M, Phillips EH, Morgenstern L, Hiatt JR (1998). The clinical spectrum of splenic infarction. Am Surg.

[B11] Franklin QJ, Compeggie M (1999). Splenic syndrome in sickle cell trait: four case presentations and a review of the literature. Mil Med.

[B12] Manes G, Kahl S, Glasbrenner B, Malfertheiner P (2000). Chronic pancreatitis: diagnosis and staging. Ann Ital Chir.

[B13] Gumbs AA (2008). Obesity, Pancreatitis, and Pancreatic Cancer. Obes Surg.

[B14] Cummings JR, Keshav S, Travis SP (2008). Medical management of Crohn's disease. Bmj.

[B15] Sarrazin J, Wilson SR (1996). Manifestations of Crohn disease at US. Radiographics.

[B16] Seidman JD, Andersen DK, Ulrich S, Hoy GR, Chun B (1991). Recurrent abdominal pain due to chronic appendiceal disease. South Med J.

[B17] Castronovo G, Ciulla A, Tomasello G, Damiani S, Maiorana AM (2006). Diverticular disease of right colon. Clinical variants and personal experience. Chir Ital.

[B18] Segura JW, Preminger GM, Assimos DG, Dretler SP, Kahn RI, Lingeman JE, Macaluso JN (1997). Ureteral Stones Clinical Guidelines Panel summary report on the management of ureteral calculi. The American Urological Association. J Urol.

[B19] Brewster DC, Cronenwett JL, Hallett JW, Johnston KW, Krupski WC, Matsumura JS (2003). Guidelines for the treatment of abdominal aortic aneurysms. Report of a subcommittee of the Joint Council of the American Association for Vascular Surgery and Society for Vascular Surgery. J Vasc Surg.

[B20] Krajka K (1973). [Clinical aspects of Yersinia enterocolitica infections]. Wiad Lek.

[B21] Farias Llamas OA, Lopez Ramirez MK, Morales Amezcua JM, Medina Quintana M, Buonocunto Vazquez G, Ruiz Chavez IE, Gonzalez Ojeda A (2005). [Peritoneal and intestinal tuberculosis: an ancestral disease that poses new challenges in the technological era. Case report and review of the literature]. Rev Gastroenterol Mex.

[B22] Salles JM, Moraes LA, Salles MC (2003). Hepatic amebiasis. Braz J Infect Dis.

[B23] Rivero Fernandez M, Moreira Vicente V, Riesco Lopez JM, Rodriguez Gandia MA, Garrido Gomez E, Milicua Salamero JM (2007). [Pain originating from the abdominal wall: a forgotten diagnostic option]. Gastroenterol Hepatol.

[B24] Krieger JN, Jacobs RR, Ross SO (2000). Does the chronic prostatitis/pelvic pain syndrome differ from nonbacterial prostatitis and prostatodynia?. J Urol.

[B25] Ness RB, Soper DE, Holley RL, Peipert J, Randall H, Sweet RL, Sondheimer SJ, Hendrix SL, Amortegui A, Trucco G (2002). Effectiveness of inpatient and outpatient treatment strategies for women with pelvic inflammatory disease: results from the Pelvic Inflammatory Disease Evaluation and Clinical Health (PEACH) Randomized Trial. Am J Obstet Gynecol.

[B26] Phillips DR, Nathanson HG, Milim SJ, Haselkorn JS (1996). Laparoscopic bipolar coagulation for the conservative treatment of adenomyomata. J Am Assoc Gynecol Laparosc.

[B27] Ortiz DD (2008). Chronic pelvic pain in women. Am Fam Physician.

[B28] Bradley CS, Nygaard IE (2005). Vaginal wall descensus and pelvic floor symptoms in older women. Obstet Gynecol.

[B29] Parente F, Cernuschi M, Antinori S, Lazzarin A, Moroni M, Fasan M, Rizzardini G, Rovati V, Morandi E, Molteni P (1994). Severe abdominal pain in patients with AIDS: frequency, clinical aspects, causes, and outcome. Scand J Gastroenterol.

[B30] McCoy HE, Kitchens CS (1991). Small bowel hematoma in a hemophiliac as a cause of pseudoappendicitis: diagnosis by CT imaging. Am J Hematol.

[B31] Jama AH, Salem AH, Dabbous IA (2002). Massive splenic infarction in Saudi patients with sickle cell anemia: a unique manifestation. Am J Hematol.

[B32] Baumgartner F, Klein S (1989). The presentation and management of the acute abdomen in the patient with sickle-cell anemia. Am Surg.

[B33] Pearigen PD (1996). Unusual causes of abdominal pain. Emerg Med Clin North Am.

[B34] Scott EM, Scott BB (1993). Painful rib syndrome – a review of 76 cases. Gut.

[B35] Karmazyn B, Steinberg R, Gayer G, Grozovski S, Freud E, Kornreich L (2005). Wandering spleen–the challenge of ultrasound diagnosis: report of 7 cases. J Clin Ultrasound.

[B36] Santoro G, Curzio M, Venco A (1990). Abdominal migraine in adults. Case reports. Funct Neurol.

[B37] Velchuru VR, Khan MA, Hellquist HB, Studley JG (2007). Eosinophilic colitis. J Gastrointest Surg.

[B38] Banikarim C, Chacko MR (2004). Pelvic inflammatory disease in adolescents. Adolesc Med Clin.

[B39] Temino VM, Peebles RS (2008). The spectrum and treatment of angioedema. Am J Med.

[B40] McOmber ME, Shulman RJ (2007). Recurrent abdominal pain and irritable bowel syndrome in children. Curr Opin Pediatr.

[B41] Korotinski S, Katz A, Malnick SD (2005). Chronic ischaemic bowel diseases in the aged–go with the flow. Age Ageing.

[B42] Brandt LJ, Boley SJ (2000). AGA technical review on intestinal ischemia. American Gastrointestinal Association. Gastroenterology.

[B43] Sreenarasimhaiah J (2005). Chronic mesenteric ischemia. Best Pract Res Clin Gastroenterol.

[B44] Otte JA, Geelkerken RH, Huisman AB, Kolkman JJ (2007). What is the best diagnostic approach for chronic gastrointestinal ischemia?. Am J Gastroenterol.

[B45] Cunningham CG, Reilly LM, Rapp JH, Schneider PA, Stoney RJ (1991). Chronic visceral ischemia. Three decades of progress. Ann Surg.

[B46] Marin Manzano E, Haurie Girelli J, Gonzalez de Olano D, Sanchez Del Corral J, Redondo Lopez S, Nunez de Arenas Baeza G, Rubio Montana M, Garcia-Prieto Bayarri MV, Utrilla Lopez A, Chinchilla Molina A (2007). [Endovascular therapy as an alternative treatment in chronic mesenteric ischemia]. Gastroenterol Hepatol.

